# Mr. J. F. Peart, F.R.C.S.I., on the Clinic Movement

**Published:** 1914-01-31

**Authors:** J. F. Peart

**Affiliations:** 10 Harley Street, W.


*To the Editor of* The Hospital.

### Mr. J. F. Peart, F.R.C.S.I., on the Clinic Movement.

*To the Editor of* The Hospital.

Sir,—I read with much interest the article on "The Clinic Movement" in your issue of January 3. I should like to endorse the writer's views as to the need for the type of clinic therein outlined, for the benefit of what might be termed the poorer middle class.

But if you will permit me I should like to point out that the need for gynæcological clinics is as urgent as that for nervous and mental disorders; perhaps, indeed, I might say more so. Under present conditions of medical education numbers of students qualify and settle down to practise without any adequate knowledge of minor gynæcological ailments or their treatment.

Moreover, for obvious reasons, from the nature of the work the number of students attending any one gynæcological clinic must be very limited, and the opportunities afforded to the students and post-graduates alike of obtaining clinical experience are very few.

When one considers, on the other hand, that the women of the struggling middle class are dependent for their health upon the general practitioners throughout the country, and that probably 50 per cent. of all women's ailments originate in, or are connected with, uterine or ovarian trouble, surely the need for proficiency amongst practitioners in the diagnosis and treatment of minor gynæcological ailments becomes apparent.

Again, this need becomes still more significant when one considers the seriousness of many uterine disorders in the later stages, the early symptoms of which had been overlooked or ignored.

Many practical questions might be asked apropos of this inability on the part of many practitioners to understand the significance of gynæcological symptoms, or to treat them properly, tending to prove the necessity for better instruction and thus the need for clinics within their reach. It would be interesting to know how many women become martyrs to the sufferings dependent on extreme degrees of prolapse, as the result of faulty treatment in the early stages; or how many cases of uterine cancer might be saved if due attention were paid by practitioners to the significance of the early symptoms.

Want of experience in making gynæcological examinations also often induces the practitioner to shirk the making of such an examination as a routine practice in the serious ailments of women, thus leading to a faulty diagnosis. An interesting example of this came under my own observation. The patient was a young woman, who had been six years married, but had never conceived. She was anxious to have children. She had been suffering for three years, and her condition had been diagnosed as one of tubercular peritonitis, in which a physician concurred. She was treated during that time for tubercular peritonitis, but no vaginal examination had been made. On examination I found she had a fibroid tumour of the uterus which was pressing on the rectum. I felt satisfied that all her symptoms could be attributed to the pelvic condition, gave a favourable prognosis for the peritonitis and sterility, and recommended removal of the fibroid. She made an uninterrupted recovery after the operation; all her abdominal symptoms disappeared, and fifteen months subsequently she gave birth to a healthy full-term child.

The fault does not always lie with the practitioner, but with the system of teaching, which does not provide adequate facilities for sufficient clinical instruction in one of the most important branches of the profession.

I sincerely trust, Sir, that this matter will be taken up by others, and result in the establishment of gynæcological clinics throughout the country.—I am, yours truly, J. F. Peart, F.R.C.S.I.

10 Harley Street, W.,

January 22, 1914.

